# The methamphetamine problem: Commentary on … Psychiatric morbidity and socio-occupational dysfunction in residents of a drug rehabilitation centre^†^

**DOI:** 10.1192/pb.bp.115.050930

**Published:** 2015-10

**Authors:** Niall Galbraith

**Affiliations:** 1 University of Wolverhampton, UK

## Abstract

This paper introduces the reader to the characteristics of methamphetamine. Explored within are the drug's effects on those who consume it as well as the history and prevalence of its use. The highly addictive nature of methamphetamine is compounded by its affordability and the ease with which it is produced, with North America and East Asia having become established as heartlands for both consumption and manufacture. The paper discusses recent cultural depictions of the drug and also the role that mental health professionals may take in designing and delivering interventions to treat methamphetamine addiction.

## The nature of methamphetamine

Methamphetamine (‘meth’) is a stimulant which increases levels of monoamines (particularly dopamine, but also noradrenaline and serotonin) in the central nervous system. Its pharmacological effects occur via a number of neurochemical processes, including disruption of vesicular^1^ and transporter^2^ functioning, through the inhibition of monoamine oxidase^3^ and the facilitation of tyrosine hydroxylase.^4^ Like other stimulants, such as cocaine and amphetamine, it produces feelings of euphoria, alertness and increased energy. Unlike cocaine though, a single dose of methamphetamine sustains these effects for many hours. Methamphetamine can be smoked, snorted, injected or swallowed. The psychological effects of long-term use include hallucinations and delusions, depression, suicidality and aggression.^5^ Withdrawal may exacerbate these symptoms, while also leading to fatigue and intense craving.^6^ Long-term health effects are considerable, and include neural damage and associated cognitive impairment,^7^ cardiovascular damage,^8^ dental disease^9^ and stroke.^10^ The drug is also associated with risky sexual behaviour, resulting in a high prevalence of sexually transmitted disease.^11^ Methamphetamine is also noted for its addictiveness. Evidence shows that addiction occurs more rapidly than with cocaine^12^ and that unlike with amphetamine, methamphetamine-seeking behaviour may persist even when tolerance is reached.^13^ The trajectory of methamphetamine use over a 10-year period has been found to resemble that of heroin more so than that of cocaine.^14^ Methamphetamine is also associated with criminality^15^ and social decline.^16^ It therefore represents a major public health, social and political dilemma.

## Who uses methamphetamine and where?

Across the world, methamphetamine use as a recreational drug has increased significantly since the 1990s, and it is reported as the second most widely misused substance, exceeded only by cannabis.^17^ In the USA during the 1960s and 1970s, methamphetamine was produced and trafficked mainly by motorcycle gangs, mostly in California.^18^ Patrons were typically White, male, blue-collar workers, but the drug has since become popular among white-collar workers, students, ethnic minorities and women,^19^ and manufacturing has spread to Midwestern states.^20^ One of the principal factors in its rise is the ease with which it can be manufactured. The chemicals necessary for its production (e.g. methylamine, ephedrine or pseudoephedrine) are relatively easy to obtain, as is the equipment required for the ‘cooking’ process. This has led to a cottage industry in methamphetamine production, with home-based laboratories being commonly uncovered by law enforcement agencies in the USA^21^ and in other parts of the world, particularly in Asia.^22^ In addition to the home lab phenomenon, there exist industrial producers of methamphetamine, who manufacture and transport large quantities of the drug.^23^ In North America, large-scale production occurs in both Mexico and Canada and the product is then brought across the border for sale within the USA. In the USA itself, 4.7% of respondents to a national survey admit to lifetime use of methamphetamine.^24^

Data from Asia also indicate high levels of use. Japan has a long history of misuse, dating back to the 1940s,^23^ when military stocks of methamphetamine flooded the market, giving rise to high incidence of misuse among young people. A second epidemic occurred in the 1970s, when use soared among blue-collar workers. This crisis has now stabilised and Japan's methamphetamine users now represent an aging population. Since the 1990s, the popularity of methamphetamine has spread to other East Asian countries. By 2007, 63% of worldwide methamphetamine seizures occurred within the Southeast Asian region, and it is estimated that half of the world's methamphetamine users are found there.^25^ The Mekong region of Myanmar, close to the border of Thailand and China, is identified as Asia's most prolific production centre for methamphetamine. From there the drug is transported across the borders for sale in neighbouring countries.^25^ In Myanmar, it is usually pressed into pill form, known colloquially as yaba (‘crazy medicine’). Thailand has suffered its own epidemic, with methamphetamine treatment admissions rising dramatically in the late 1990s,^26^ but evidence of increasing methamphetamine use is also found in Brunei, Laos, the Philippines^22^ and Cambodia.^27^

In Europe, the meth epidemic has not yet arrived, perhaps because there is already a congested market for stimulant drugs, although the Czech Republic and to a lesser extent Slovakia have a history of high methamphetamine use.^28^ In Australia, use has increased in recent years but not dramatically.^29^ In South Africa, the past decade has seen a significant increase in treatment admissions for methamphetamine.^30^ This increase in methamphetamine use is positively associated with risk-taking sexual behaviour,^31^ which if unchecked may in turn exacerbate an already urgent HIV epidemic.

## Cultural depictions of methamphetamine

The emergence of methamphetamine as one of the most widely used recreational drugs is associated with its rise in the media. Methamphetamine has become a cultural phenomenon, in much the same way that heroin, MDMA (contracted from 3,4-methylenedioxy-methamphetamine; ecstasy) and cannabis had become popularised already. The most obvious cultural reference to methamphetamine is in the hugely successful American drama series *Breaking Bad*. This drama describes the exploits of a terminally ill chemistry teacher who chooses to become a manufacturer and then seller of methamphetamine, initially to guarantee financial security for his family after his death. The series focuses on the corruption of the main character and the erosion of his relationships with those close to him. What is notable about the series though is that the problem of devastating effects of methamphetamine on individuals and communities occupies only a minor part in the story. The series has done much to publicise the existence of methamphetamine to households across the world, but in not fully exploring its sinister effects (other than the moral degeneration of those who manufacture it), the series runs the risk of sanitising or normalising this destructive drug to the wider society.

At the other extreme, also in the USA, there has been a widely publicised campaign to highlight the unpleasant physical effects of methamphetamine addiction. The ‘Faces of Meth’ project^32^ exposes police custody photographs of users, showing images of the same individual at different points in time, so as to longitudinally chronicle the ravages of the drug on physical appearance. These before and after photos – which reveal apparently common features of long-term methamphetamine use: skin damage (caused by obsessive picking) and dental ill health (or ‘meth mouth’ as it is colloquially known)^33^ – are designed to shock and appal observers. The effectiveness of the scheme is difficult to assess due to the absence of trials, however, the use of fear and shock is not always an effective deterrent in health campaigns and is generally regarded as inferior to positive reinforcement approaches.^34^

The Faces of Meth-type approach has come under criticism from Naomi Murakawa,^35^ who argues that its focus on the visual effects of methamphetamine, mostly in White methamphetamine users, represents a type of social panic. Murakawa argues that historically, drug panics in the USA have been constructed in line with racial prejudices (e.g. Chinese-focused opium scares, Mexican-focused cannabis scares and Black-focused crack scares). Methamphetamine addiction is often described along racial lines as a ‘White trash’ phenomenon. Murakawa claims that decayed or missing teeth mark prevailing fears over the decline in White social status, as traditional representations of American so-called ‘White trash’ typically depict poor dental health as a visual indicator of lower class.

Given the prevalence of methamphetamine use across the globe, considerable effort has been put into designing effective treatment programmes for its users. Broadly speaking, these interventions are pharmacological, psychosocial or community-based prevention approaches. The evidence in favour of pharmacological treatments is mixed, although some promising findings with modafinil, bupropion and naltrexone have been reported.^36^ Psychosocial interventions have proved effective in the short term, but more evidence is needed to demonstrate long-term benefits.^37^ Community-based prevention schemes have also shown evidence of benefit.^38^ The promise shown by such interventions is encouraging, given the addictiveness of methamphetamine, the intensity and duration of cravings experienced by those who go through withdrawal^6^ and also the psychological comorbidity. Interestingly for mental health professionals, there is evidence that the cost-effectiveness of treatment^39^ and prevention^38^ approaches may compare favourably with alternatives, such as, for example, interventions by law enforcement to disrupt the supply of the precursor chemicals needed for methamphetamine production.^40^ Furthermore, given the advance of this drug across Asia and North America and its potential for expansion across thus far untapped markets (e.g. Europe and Africa), the further development of robust treatment programmes for the future is urgently needed.
